# Association between intestinal functional disorders and anal fistula: evidence from a retrospective case–control study

**DOI:** 10.7717/peerj.21466

**Published:** 2026-06-26

**Authors:** Rui Zhang, Huixiang Li, Hanlin Gong

**Affiliations:** Department of Integrated Traditional Chinese and Western Medicine, West China Hospital of Sichuan University, Chengdu, Sichuan, China

**Keywords:** Bowel dysfunction, Diarrhea, Anal fistula, Association, Retrospective study

## Abstract

**Background:**

Clinical observations indicate that some individuals experience bowel dysfunction, such as diarrhea or constipation, prior to the diagnosis of anal fistula (AF). These observations suggest a potential association between pre-existing intestinal dysfunction and the occurrence of AF. However, systematic clinical evidence remains limited. This study aimed to evaluate the association between pre-diagnostic bowel dysfunction and AF and to explore whether such associations differ across AF anatomical subtypes.

**Methods:**

We conducted a retrospective hospital-based case-control study including 251 patients with newly diagnosed AF between July 2024 and July 2025. During the same period, 251 age- and sex-matched individuals without AF were selected as controls (1:1 matching; age ± 3 years). Demographic characteristics, lifestyle factors, and bowel function within 1 year before the index date were collected from medical records and supplementary interviews where applicable. Conditional logistic regression, accounting for the matched-pair design, was used to assess the association between bowel dysfunction and AF. Both crude (unadjusted) and adjusted analyses were performed. Stratified analyses were performed to explore heterogeneity across AF subtypes.

**Results:**

Pre-existing bowel dysfunction was associated with a higher likelihood of developing AF (*p* < 0.05). Diarrhea showed a significant association with AF (OR ≈ 2.2), whereas constipation was not significantly associated. Smoking, alcohol consumption, prolonged sedentary or standing occupations, and higher body mass index (BMI) were also associated with AF. In exploratory analyses of AF subtypes, diarrhea showed modest heterogeneity across anatomical classifications, whereas constipation showed no significant variation across subtypes.

**Conclusions:**

This retrospective case-control study indicates that pre-existing intestinal functional disorders, particularly chronic diarrhea, are associated with a higher likelihood of AF, with the association appearing more pronounced among low anal fistulas. No consistent subtype-specific pattern was observed overall, although diarrhea showed modest heterogeneity across anatomical classifications in exploratory analyses. Given the retrospective observational design, these findings support association rather than causation and warrant confirmation in multicenter prospective studies. Clinically, these results suggest that earlier identification and risk assessment of patients with chronic diarrhea and other intestinal functional disturbances may provide valuable insights, but further research is needed to confirm these findings.

## Introduction

Anal fistula (AF) is a common anorectal disorder characterized by an abnormal epithelialized tract connecting the anal canal or rectum to the perianal skin, typically comprising an internal opening, fistulous tract, and external opening (Włodarczyk et al., 2021). From a clinical perspective, AF commonly presents with perianal inflammation, including swelling, erythema, and pruritus. Patients may also report symptoms such as purulent discharge, persistent pain, and occasionally bleeding.

Epidemiological studies suggest that the incidence of AF has increased worldwide, with an estimated annual rate of 1–2 cases per 10,000 individuals. AF represents approximately 8–25% of all anorectal diseases. The condition predominantly affects individuals between 30 and 50 years, with peak incidence observed around 40 years of age. It also shows a male-to-female ratio of approximately 2:1 (Perregaard et al., 2021). AF are commonly classified according to their relationship to the sphincter complex, most classically by the Parks classification, which includes intersphincteric, transsphincteric, suprasphincteric, and extrasphincteric fistulas (Parks, Gordon & Hardcastle, 1976). Simple fistulas typically encompass intersphincteric and low transsphincteric tracts, affecting <30% of the external anal sphincter. In contrast, complex fistulas are characterized by high transsphincteric, suprasphincteric, extrasphincteric, and horseshoe fistulas (Bubbers & Cologne, 2016).

It is widely accepted that chronic suppurative infection, resulting in epithelial disruption, plays a central role in the pathogenesis of AF (Scharl & Rogler, 2014). Two main hypotheses have been proposed: the anal gland infection theory and the central space infection theory (Włodarczyk et al., 2021). The former theory considers AF as a sequela of perianal abscesses, wherein obstruction and secondary infection of the anal glands result in abscess formation that traverses the sphincter complex and eventually forms a fistulous tract (Amato et al., 2020). The latter theory, proposed by Shafik (1987), postulates that pathogens originate from the compromised epithelium of the anal canal rather than the anal crypts. This process initiates the formation of a central abscess, which subsequently spreads outward (Garg, 2019). In addition, AF may occur secondary to specific disorders, most notably inflammatory bowel disease (IBD) such as Crohn’s disease (Abbas, Jackson & Haigh, 2011; McGregor, Tandon & Simmons, 2023). More recently, attention has also shifted toward host and molecular factors, including immune dysregulation (Litta et al., 2022), cytokine signaling, epithelial-mesenchymal transition, and other noninfectious mechanisms that may contribute to fistula development or persistence (Andreou et al., 2020). Nevertheless, clinical experience indicates that infection-centered frameworks alone may not fully explain inter-individual susceptibility or the heterogeneity of AF presentation.

Notably, some patients report long-standing bowel dysfunction, particularly chronic diarrhea or constipation, preceding AF diagnosis. Such pre-diagnostic bowel dysfunction is biologically plausible as a correlate of AF occurrence through both local and systemic pathways. Locally, chronic diarrhea may increase perianal moisture and maceration, facilitate repeated epithelial microtrauma due to frequent stools, and promote bacterial ingress, thereby amplifying crypt inflammation and abscess formation. Constipation may plausibly contribute to repeated straining, local mucosal trauma, and impaired anorectal hygiene, which could theoretically increase susceptibility to local inflammation (Schlichtemeier & Tou, 2018; Seifarth et al., 2021). Beyond these mechanical and local inflammatory mechanisms, bowel dysfunction may also reflect broader gastrointestinal perturbations, including intestinal dysbiosis, impaired mucosal barrier integrity, and enteropathy-related epithelial vulnerability. These processes can promote microbial translocation and immune activation, potentially shaping inflammatory tone and tissue repair capacity in distal mucosal sites, thereby offering a biological context linking bowel symptoms to anorectal infectious complications. Importantly, these mechanistic considerations remain hypothesis-generating in most clinical observational settings and require validation using objective biomarkers and microbiome-related assessments.

Despite increasing clinical attention, existing literature on AF has largely focused on postoperative outcomes, surgical techniques, and local anorectal factors, whereas evidence regarding pre-existing bowel dysfunction as a correlate of AF occurrence remains limited. Moreover, whether bowel dysfunction is associated with AF anatomical classification has not been adequately characterized. To address these gaps, we conducted a retrospective case–control study to examine whether bowel dysfunction within a predefined exposure window is associated with AF, and to explore potential heterogeneity across AF subtypes while adjusting for major confounders. Given the observational and retrospective design, our analyses were intended to evaluate associations rather than infer causality. These findings may help refine clinical risk stratification and motivate future prospective studies incorporating objective gastrointestinal evaluation and mechanistic measurements to clarify underlying pathways.

## Materials & Methods

### Study design

A retrospective single-center case-control study was conducted at the Department of Integrated Traditional Chinese and Western Medicine, West China Hospital, Sichuan University, between July 2024 and July 2025. Given the retrospective design, the study is inherently vulnerable to selection bias and information bias. In particular, part of the exposure information was supplemented using structured telephone interviews when medical records were incomplete, which may introduce recall bias. Trained physicians extracted data from electronic medical records (EMR), and missing or ambiguous items were supplemented *via* telephone interviews conducted by two trained investigators using a standardized script and prespecified definitions of diarrhea and constipation, with responses recorded on structured case report forms. The exposure window was standardized and anchored to the index date (within 1 year prior to diagnosis for cases and within 1 year prior to the matched index date for controls). Interviewers were not informed of the study hypothesis and followed identical probing procedures for cases and controls to minimize differential ascertainment. To reduce misclassification, EMR-documented diagnoses and medication use were prioritized when discrepancies arose, and discordant records were independently reviewed by two investigators and resolved by consensus. For the retrospective chart review component, the requirement for written informed consent was waived by the Institutional Review Board. When supplementary telephone interviews were conducted to clarify missing information, verbal consent for the interview was obtained at the beginning of each call.

### Participants

#### •  Case group

Inclusion criteria: Patients were considered eligible if they had a first-time AF diagnosis, had ≥12 months of medical records before diagnosis, and had sufficient documentation of bowel symptoms and relevant clinical variables.

Exclusion criteria: Patients were excluded if they had a history of inflammatory bowel disease (IBD), colorectal tumors, spinal cord injury, tuberculosis, recent chemotherapy or radiotherapy, prior perianal surgery, psychiatric illness, pregnancy, or incomplete records.

#### •  Control group

Inclusion criteria: Controls were individuals with no current or prior history of AF or other documented proctologic disorders during the same period, who had ≥12 months of available medical records, and were successfully matched to cases by sex and age (±3 years).

Exclusion criteria: Patients were excluded if they had a history of IBD, colorectal tumors, spinal cord injury, tuberculosis, recent chemotherapy or radiotherapy, prior perianal surgery, psychiatric illness, pregnancy, or incomplete records.

### Data collection and matching

To ensure data accuracy, clinical data were independently extracted from EMR by two independent physicians, with any discrepancies resolved through consensus and report agreement (kappa/ICC). Missing or ambiguous information was supplemented *via* structured telephone interviews.

For each AF case, one control was selected from the same clinical department and within the same study period (July 2024–July 2025). Eligible controls included hospitalized patients or outpatients without a confirmed diagnosis of AF. To minimize confounding bias, individual 1:1 matching was performed based on sex and age (±3 years), thereby ensuring comparability of baseline demographic characteristics between the two groups. Controls were selected from the same department and study period as cases to enhance comparability and reduce differential healthcare access. Cases with missing data on key exposure, outcome, or matching variables were excluded from the corresponding analyses. Nevertheless, as a hospital-based study, Berkson’s bias and limited generalizability to the community population cannot be excluded.

### Definition of anal fistula

AF was diagnosed according to the Traditional Chinese Medicine Industry Standards of the People’s Republic of China (ZY/T001.7-94). Diagnosis was then confirmed through clinical manifestations, physical examination, and imaging, including fistulography and MRI, in accordance with the ICD-10 codes K60.3 (AF) and K60.4 (complex AF). Typical diagnostic features of AF comprised purulent discharge, pain, and pruritus. Further classification was based on fistula tract anatomy and the number of external openings, distinguishing cases as low *versus* high, and simple *versus* complex.

(a) Low AF: tract located below the deep layer of the external anal sphincter, internal opening at the crypt.

(b) High AF: tract located above the deep layer of the external anal sphincter, internal opening at the crypt.

(c) Simple AF: single, straight tract with one internal or external opening, involving only a portion of the sphincter.

(d) Complex AF: multiple or branching tracts, involving deep sphincter layers, with ≥2 openings or associated blind tracts.

### Definition of intestinal dysfunction

Bowel dysfunction was operationally defined for this study using symptom-based clinical criteria and the Bristol Stool Form Scale within the 12 months preceding the index date.

• Diarrhea:

Defined according to the Textbook of Internal Medicine (8th Edition). Patients were classified as having diarrhea if they met ≥1 of the following:

(a) ≥25% of stools loose or watery;

(b) Defecation frequency ≥21 times/week (≥3/day);

(c) Bristol stool scale type 6–7;

(d) Chronic duration ≥4 weeks.

• Constipation:

Defined as ≥1 of the following:

(a) Straining during ≥25% of defecations;

(b) Passage of hard stools in ≥25% of defecations (<3/week);

(c) Sensation of incomplete evacuation in ≥25% of defecations;

(d) Sensation of anorectal obstruction in ≥25% of defecations;

(e) Manual maneuvers required in ≥25% of defecations;

(f) Bristol stool scale type 1–2.

Associated symptoms included straining, anorectal blockage, or need for manual assistance.

### Outcome measures

The index date was defined as the first clinical encounter for AF between July 2024 and July 2025. A retrospective review was conducted to determine bowel dysfunction status within 1 year before the diagnosis. Data were collected through medical records and supplemented by outpatient, inpatient, or telephone interviews when necessary. The variables collected included demographics (age, sex, BMI, and occupation), lifestyle factors (smoking, alcohol consumption, and high-fat diet), comorbid conditions, bowel function status, and the specific subtype of AF. Records lacking critical data elements were excluded from the analysis to ensure the integrity and completeness of the dataset.

**Figure 1:**
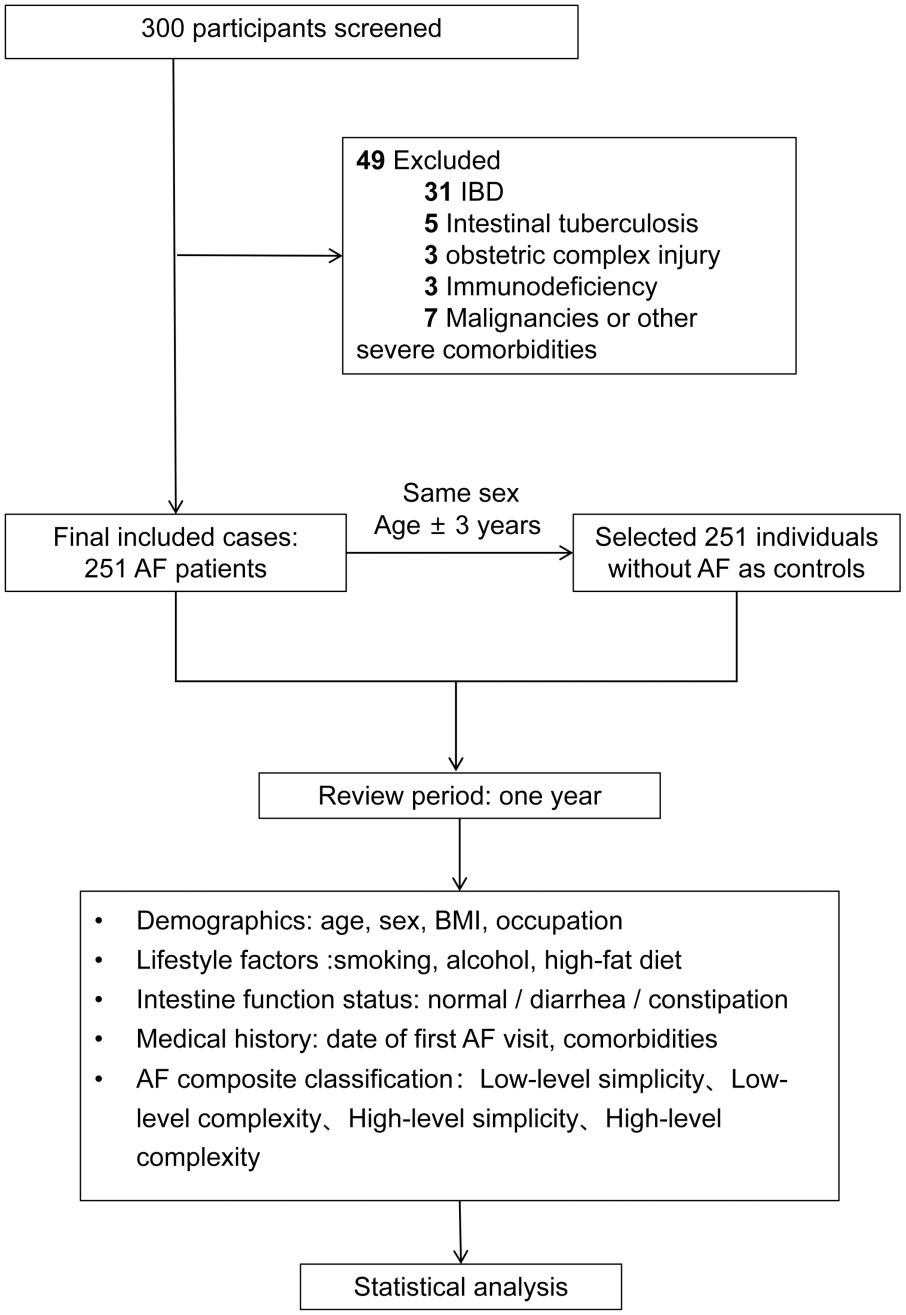
Flowchart.

Variables included in the multivariable model were selected *a priori* based on clinical relevance and data completeness, including BMI, smoking, alcohol consumption, sedentary/standing occupation, and high-fat diet. Several potentially relevant variables (*e.g.*, diabetes, recent antibiotic exposure, prior gastrointestinal infection, and use of stool-regulating medications) were not consistently available in the retrospective records and therefore were not included in the adjusted model (Fig. 1).

### Ethics

This retrospective study involved the review of existing clinical records from July 2024 to July 2025, using de-identified data. Data extraction and analysis for research purposes were initiated only after obtaining approval from the Institutional Review Board (IRB) (Approval No. 2025 (1971)), with written informed consent waived due to the use of de-identified data. However, verbal consent was obtained at the beginning of each supplementary telephone interview conducted to clarify missing information. The study protocol was approved by the Biomedical Research Ethics Committee of West China Hospital of Sichuan University, in accordance with the Declaration of Helsinki and applicable privacy regulations.

### Statistical analysis

All statistical analyses were performed using SPSS software version 22.0 (IBM Corp., Armonk, NY, USA). Two-sided tests were applied, and a *p*-value < 0.05 was considered statistically significant. Continuous variables were summarized as mean ± standard deviation (SD), and categorical variables were presented as counts and percentages. Baseline characteristics were described for cases and controls to assess overall comparability. Because cases and controls were individually matched 1:1 on age (±3 years) and sex, the association between bowel dysfunction and AF was estimated using conditional logistic regression stratified by matched pairs. Odds ratios (ORs) and 95% confidence intervals (CIs) were calculated. Both crude and adjusted analyses were performed within the matched-pair framework. The crude analysis assessed the association between bowel dysfunction and AF without adjustment for additional covariates. The adjusted analysis controlled for BMI, smoking status, alcohol consumption, sedentary or prolonged standing occupation, and high-fat diet. Participants with missing or invalid BMI data were excluded from analyses involving BMI. The adjusted model was based on a complete-case analysis. Exploratory subgroup analyses were conducted according to AF anatomical subtype to assess possible heterogeneity in the association between bowel dysfunction and AF. Given the limited sample size within some subgroups, these analyses were considered exploratory and were interpreted cautiously.

## Results

A total of 300 patients diagnosed with AF were initially screened. Patients with comorbidities, such as IBD, tuberculosis, radiation injury, malignant fistula, obstetric complex injury, and immunodeficiency, were excluded. After removing 49 ineligible cases, 251 patients with AF were retained for analysis. A control cohort of 251 individuals without AF was subsequently enrolled using 1:1 matching based on sex and age (±3 years). Altogether, 502 participants were included in the final analysis.

### Demographic characteristics

The case group comprised 195 males and 56 females, corresponding to a male-to-female ratio of approximately 3.5:1, in line with the established male predominance of AF. Patient age ranged from 14 to 73 years, with a mean of 39.3 ± 12.0 years. In males, the age range was 14–73 years (mean 39.3 ± 11.7 years), whereas in females, it was 18–72 years (mean 39.4 ± 13.0 years). Comparative analysis revealed no significant differences between the case and control groups in baseline characteristics, including age (*p* = 0.864), sex (*p* = 1.000), and high-fat dietary habits (*p* = 0.730) (Table 1). These findings confirm good matching and comparability between the groups.

**Figure 2:**
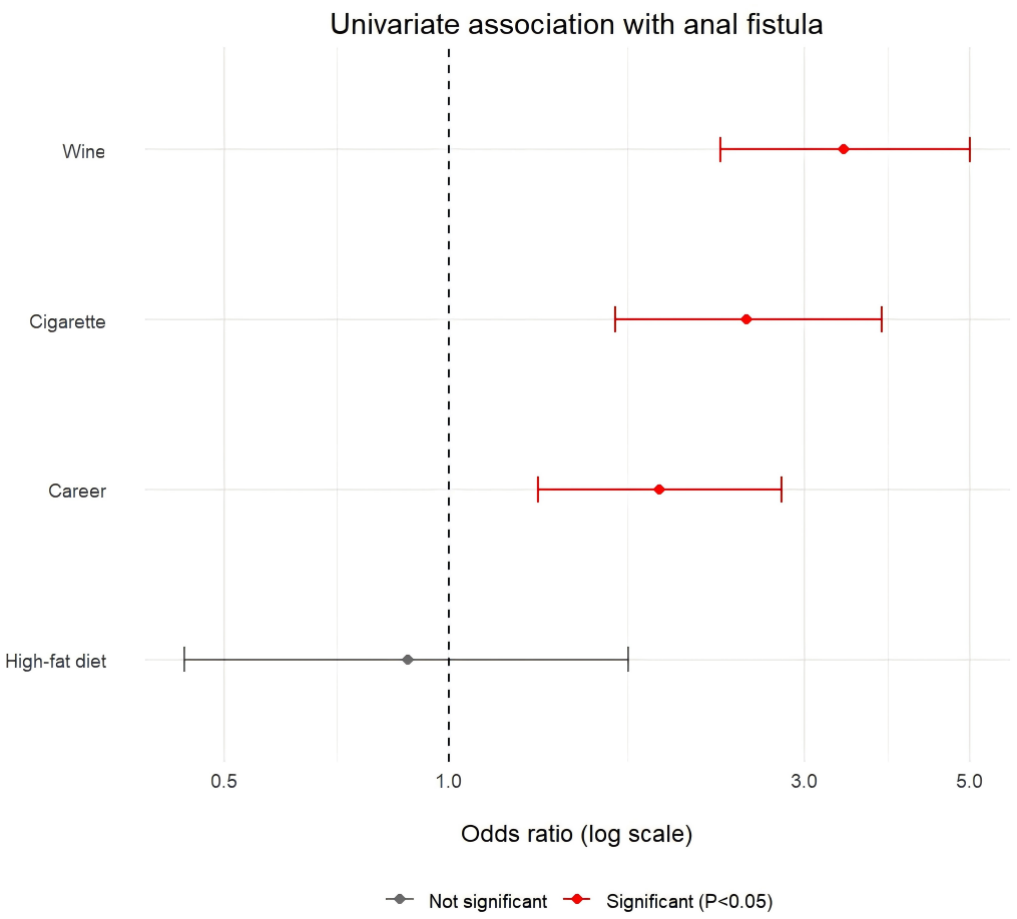
Univariate association with anal fistula.

However, the case group demonstrated significantly higher BMI than the control group (*p* < 0.001). Lifestyle analysis further revealed that smoking (*p* < 0.001), alcohol consumption (*p* < 0.001), and sedentary occupation (*p* < 0.001) were all significantly more prevalent among cases than controls. ORs and 95% CIs were estimated to test the hypothesis that alcohol consumption, smoking, and occupations involving prolonged sedentary or standing postures are linked to a higher likelihood of AF, whereas high-fat dietary habits showed no significant association (Table 2 and Fig. 2).

### Association between intestinal dysfunction and AF occurrence

**Figure 3:**
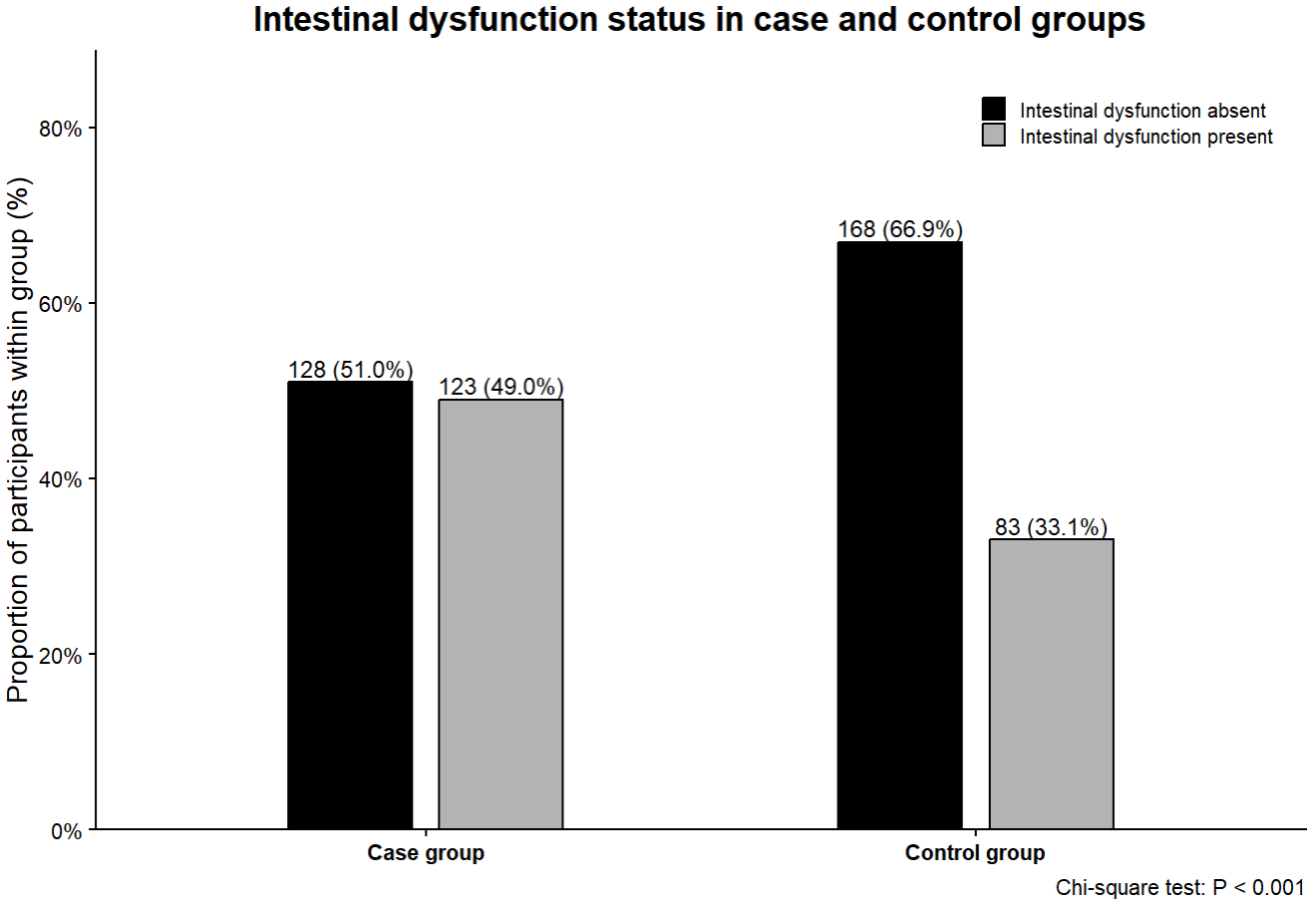
Intestinal dysfunction status in case and control groups.

Pre-existing bowel dysfunction was observed in 49.0% (123/251) of the case group and 33.1% (83/251) of the control group, with a statistically significant between-group difference (Fig. 3). This finding suggests a potential association between AF and antecedent bowel dysfunction.

In case–control analysis, pre-diagnostic diarrhea was reported in 28.7% of AF patients, a prevalence significantly higher than the 14.3% reported in the control group (*p* < 0.001). In contrast, the pre-existing constipation was reported in 20.3% of cases and 18.7% (47/251) of controls, with no significant between-group difference (*p* = 0.736) (Table 3). This finding suggests that constipation may not be strongly associated with AF.

### Distribution types of AF in the case group

**Figure 4:**
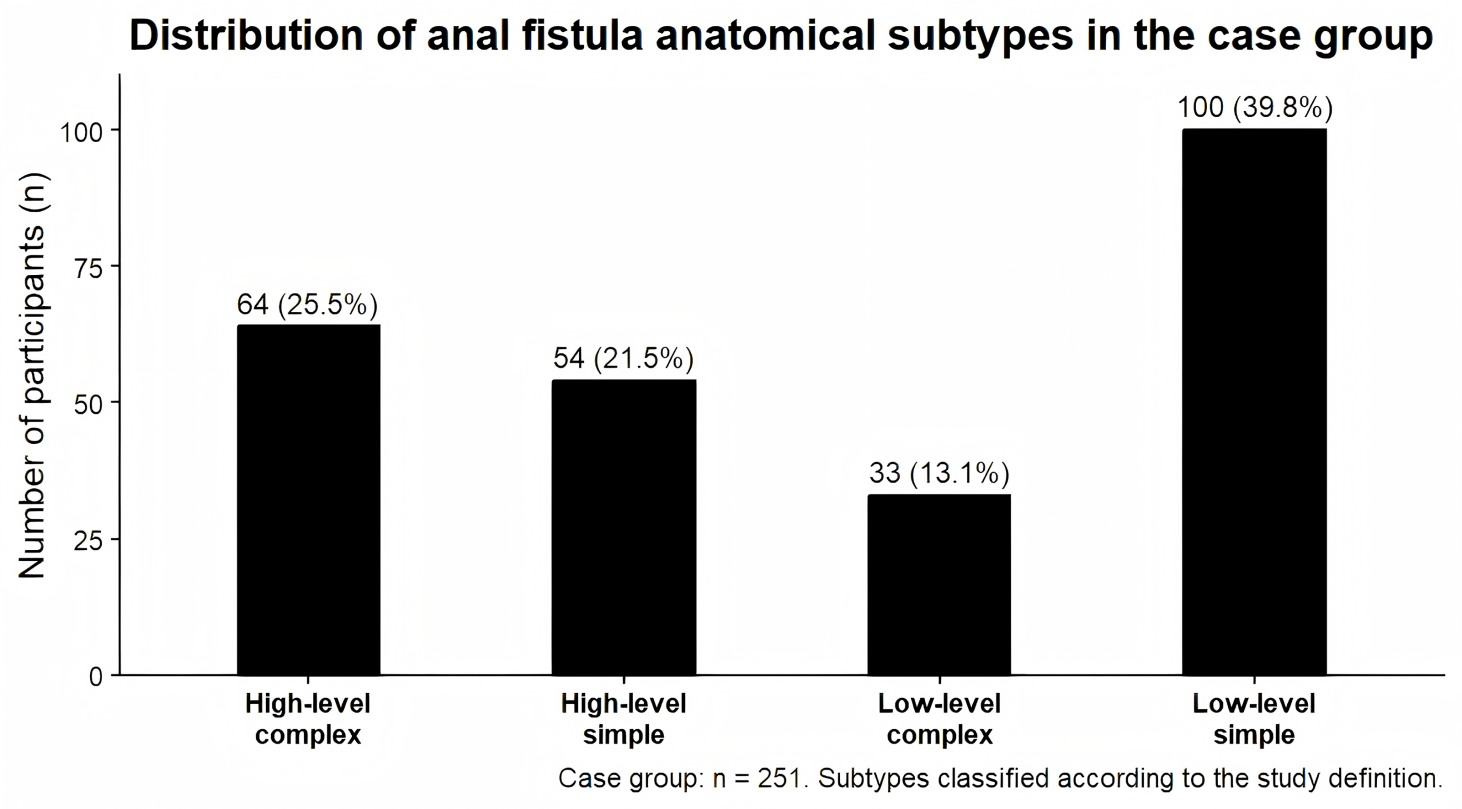
Distribution of anal fistula anatomical subtypes in the case group.

Among the 251 patients with AF, low-level fistulas constituted the predominant form (*n* = 133), surpassing high-level fistulas (*n* = 118). Based on complexity, 154 cases (61.4%) were classified as simple fistulas, whereas 97 cases (38.6%) were defined as complex fistulas. Combined subtype analysis demonstrated that low-level simple fistulas were the most frequent subtype (*n* = 100, 39.8%), followed by high-level complex (*n* = 64, 25.5%), high simple (*n* = 54, 21.5%), and low complex fistulas (*n* = 33, 13.1%) (Fig. 4). Collectively, low-level and simple fistulas predominated in the case group, indicating that AF more often manifests as superficial lesions with relatively uncomplicated anatomical structures.

**Table 1:** Demographic characteristics of the case group and control group.

**Factors**	**Case group**	**Control group**	***p* value**
Age (mean (SD))	39.3 ± 11.8	39.4 ± 12.1	0.864
Sex (n (%))			
Male, n (%)	195 (77.7%)	195 (77.7%)	1.000
Female, n (%)	56 (22.3%)	56 (22.3%)	1.000
BMI^1^, mean ± SD	24.8 ± 4.0	23.7 ± 3.0	<0.001
Smoking, n (%)	91 (36.3%)	46 (18.3%)	<0.001
Alcohol consumption, n (%)	126 (50.2%)	57 (22.7%)	<0.001
Sedentary occupation, n (%)	105 (41.8%)	68 (27.1%)	<0.001
High-Fat Diet, n (%)	17 (6.8%)	19 (7.6%)	0.73

**Notes.**

1 BMI, Body mass index.

**Table 2:** Univariate Odds Ratios for Factors Associated with AF.

**Factors**	**OR**	**95% CIs**	***p* value**
Alcohol consumption	3.43	(2.33–5.04)	<0.001
Smoking	2.53	(1.68–3.82)	<0.001
Sedentary or prolonged standing occupation	1.94	(1.33–2.82)	<0.001
High-fat diet	0.89	(0.45–1.75)	0.73

### Logistic regression analysis of intestinal dysfunction and odds of AF

In conditional logistic regression, both crude and adjusted analyses followed the matched-pair framework. pre-existing bowel dysfunction was associated with higher odds of AF (OR = 2.03, 95% CI [1.38–2.97]; *p* < 0.001). Among the specific bowel dysfunction categories, diarrhea was significantly associated with AF (OR = 2.33, 95% CI [1.49–3.66]; *p* < 0.001), whereas constipation was not (OR = 1.11, 95% CI [0.71–1.74]; *p* = 0.647) (Table 4). Crude analysis, without adjustments for any confounders, showed similar results, with diarrhea significantly associated with AF (OR = 2.45, 95% CI [1.56–3.84]; *p* < 0.001). After adjusting for BMI, smoking, alcohol consumption, sedentary/standing occupation, and high-fat diet, diarrhea remained significantly associated with AF (adjusted OR = 1.84, 95% CI [1.24–2.72], *p* = 0.002).

### Relationship between types of AF and intestinal dysfunction

In subgroup analysis, diarrhea was more common in low-level AF than in high-level AF (36.1% *vs.* 20.3%, *p* = 0.009). However, diarrhea did not differ significantly between simple and complex AF cases (29.9% *vs.* 26.8%, *p* = 0.704) (Table 5). Constipation showed no significant variation across subtype groupings when classifications were collapsed into low *vs* high (21.1% *vs.* 19.5%, *p* = 0.881) or simple *vs* complex categories (21.4% *vs.* 18.6%, *p* = 0.697) (Table 6).

**Figure 5:**
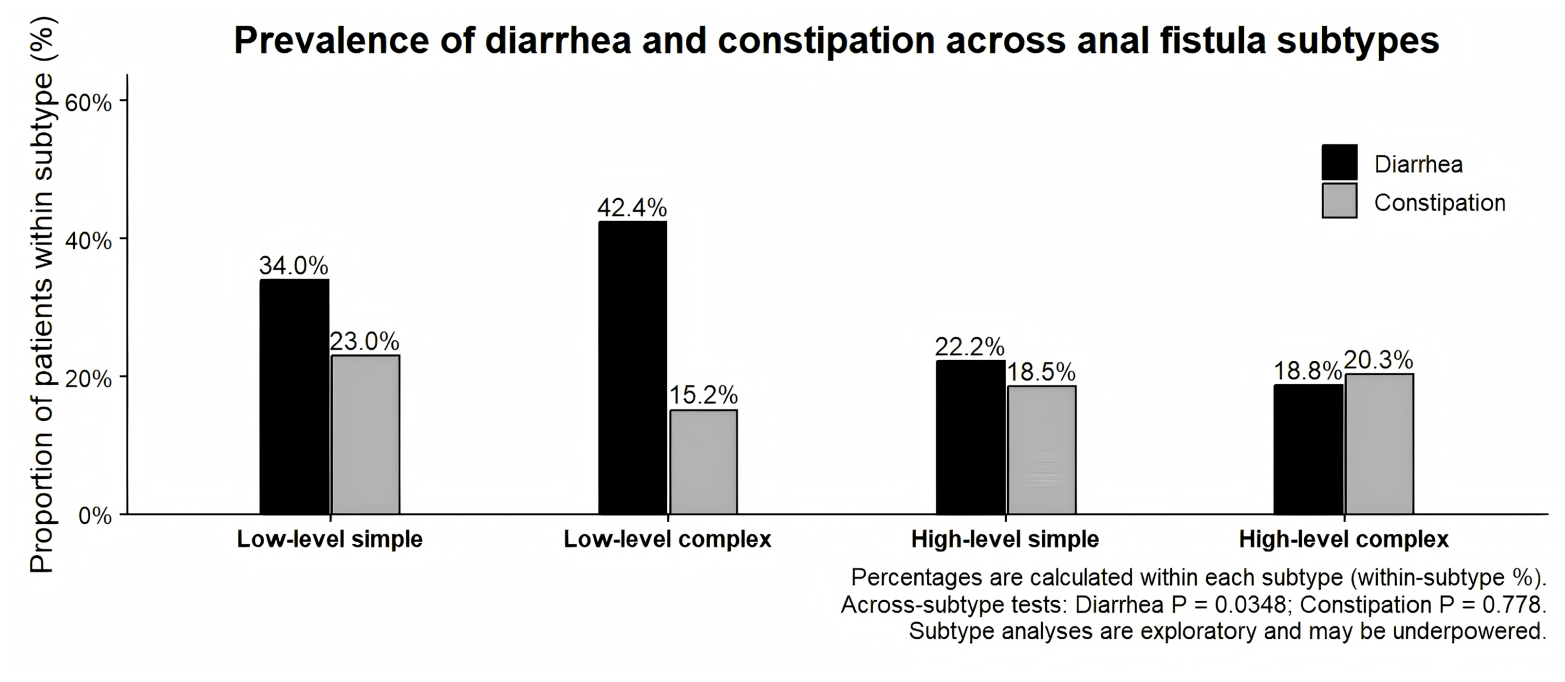
Prevalence of diarrhea and constipation across anal fistula subtypes.

When further stratified into four anatomical subtypes (low simple, low complex, high simple, and high complex), the prevalence of diarrhea showed modest heterogeneity (34.0%, 42.4%, 22.2%, and 18.8%, respectively; *p* = 0.0348), while constipation did not (23.0%, 15.2%, 18.5%, and 20.3%, respectively; *p* = 0.778) (Fig. 5). Given the exploratory nature of these subgroup analyses and the limited sample sizes within some subgroups, these findings should be interpreted cautiously.

### Stratified analysis

A total of 497 participants (after excluding five with missing or invalid BMI data) were included in this stratified analysis. Stratified analysis revealed a significant association between higher BMI and the likelihood of developing AF. Overweight patients (BMI = 25–29.9) exhibited a 1.57-fold higher odds of AF occurrence (OR = 1.57) than individuals with normal BMI (18.5–24.9). This risk was further amplified in the obese group (BMI ≥ 30), with an approximate threefold increase (OR = 2.96). In contrast, underweight patients (BMI < 18.5) did not show a statistically significant difference compared with the reference group (OR = 1.13, 95% CI [0.44–2.86], *p* = 0.801) (Table 7). Subgroup analyses were exploratory and were not powered to detect modest between-subtype differences. Therefore, the subgroup findings should be interpreted cautiously.

**Table 3:** Comparison of diarrhea and constipation incidence between the two groups.

**Intestinal dysfunction**	**Case group (*n* = 251)**	**Control group (*n* = 251)**	***p* value** ^a^
Diarrhea	72 (28.7%)	36 (14.3%)	<0.001
Constipation	51 (20.3%)	47 (18.7%)	0.736

**Notes.**

a *p* values were calculated using Fisher’s exact test (two-sided) for between-group comparisons of proportions.

## Discussion

Due to the retrospective case–control design, our findings suggest associations rather than causal effects. This study demonstrated a significant link between pre-existing bowel dysfunction and increased likelihood of AF, independent of BMI and lifestyle-related factors.

Chronic diarrhea exhibited the strongest association with AF. Plausible explanations include (a) sustained perianal moisture facilitating pathogen adhesion and proliferation; (b) dysbiosis and recurrent cryptoglandular infection sustaining local inflammation; and (c) increased defecatory frequency and altered intestinal motility leading to repeated stimulation of the anal crypts, thereby augmenting opportunities for infection. By contrast, constipation likely acts mainly through mechanically induced trauma from hard stools and obstruction of the anal crypts. However, in the present study its effect was weaker and did not reach statistical significance, possibly owing to limited sample size, the operational definition of constipation, and stratification schema. Confirmation in larger cohorts with refined phenotyping is warranted.

**Table 4:** Conditional logistic regression analysis of intestinal dysfunction and AF odds (matched pairs).

**Exposure factors**	OR (95% CI)^*^	***p* value** ^a^
Intestinal dysfunction	2.03 (1.38–2.97)	<0.001
Constipation	1.11 (0.71–1.74)	0.647
Diarrhea	2.33 (1.49–3.66)	<0.001

**Notes.**

* ORs are presented for presence vs. absence of the exposure (reference = no dysfunction, no constipation, no diarrhea).

a Conditional logistic regression stratified by 1:1 matched pairs (matched on age ±3 years and sex); *p* values derived from Wald tests.

**Table 5:** Comparison of diarrhea occurrence among patients across various types of AF.

**AF classification**	**Patients with diarrhea, n/N (%)**	**x** ^**2**^ **/*p* value**
**Level**		
Low	48/133 (36.1%)	*x*^2^ = 6.9, *p* = 0.009
High	24/118 (20.3%)	
**Complexity**		
Simple	46/154 (29.9%)	x^2^≈ 0.14, *p* = 0.704
Complex	26/97 (26.8%)	

**Table 6:** Comparison of constipation occurrence among patients across various types of AF.

**AF classification**	**Patients with constipation, n/N (%)**	**x** ^**2**^ **/*p* value**
**Level**		
Low	28/133 (21.1%)	x^2^≈ 0.02, *P* = 0.881
High	23/118 (19.5%)	
**Complexity**		
Simple	33/154 (21.4%)	x^2^≈ 0.15, *P* = 0.697
Complex	18/97 (18.6%)	

**Table 7:** Logistic regression analysis of BMI categories and the odds of AF.

**BMI category (kg/m** ^**2**^ **)**	**OR (95% CI)**	***P* value**
18.5–24.9 (Ref.)	1.00 (Ref.)	–
<18.5	1.13 (0.44–2.86)	0.801
25–29.9	1.57 (1.06–2.32)	0.023
≥ 30	2.96 (1.41–6.22)	0.004

**Notes.**

* Analyses are based on 497 participants after excluding 5 with missing or invalid BMI data.

a Unconditional logistic regression analysis adjusted for age and sex.

Diarrhea showed modest heterogeneity across subtypes, whereas constipation did not vary significantly. However, given the limited statistical power, these findings should be interpreted with caution. After AF forms, its complexity and tract course are more likely shaped by the spread of infection and surrounding anatomical structures than on bowel habits per se. In addition, body mass index (BMI) exhibited a dose–response relationship with AF risk; obesity-related chronic low-grade inflammation, metabolic dysregulation, immune imbalance, and gut dysbiosis may facilitate infection and recurrent inflammation, thereby contributing to pathogenesis. However, among patients with established AF, the presence of diarrhea or constipation did not vary meaningfully across various subtypes or tract patterns. This observation suggests that bowel dysfunction is primarily associated with AF occurrence, but showed no clear association with subtype pattern.

Due to the inherent anatomical vulnerability of the anorectal region, bowel dysfunction may be an important correlate of AF in clinical practice. Abnormal defecation can cause fecal stasis within the anal crypts, thereby facilitating localized infection and inflammatory responses (Mujukian et al., 2020). Such dysfunction may also alter luminal pH, disrupt the integrity of the mucosal barrier, and impair intestinal motility, thereby further increasing infection risk (Amato et al., 2020). Moreover, extrinsic factors, such as antibiotic misuse and dietary imbalances, can disrupt the “brain–gut–microbiota” axis, further exacerbating bowel dysfunction (Mei et al., 2019). These mechanistic explanations are hypothesis-generating because microbiome profiles and barrier-related biomarkers were not assessed in our study.

Currently, the front-line management of AF still primarily relies on surgery, yet its efficacy is limited. High and complex AF, particularly those involving the levator ani or external anal sphincter, represent some of the most formidable challenges in anorectal surgery (Arroyo et al., 2020; Celayir et al., 2020). While traditional operations and emerging techniques, such as rectal advancement flap and fibrin glue injection (Rosenbaum, Vetterlein & Fisch, 2020), can effectively remove fistula tracts and promote healing, they are often associated with postoperative complications and functional impairment. These issues, along with high recurrence rates, can substantially impair patients’ quality of life.

Taken together, these findings suggest that, in addition to standard surgical management, greater attention to upstream risk assessment and bowel-function-related factors may be clinically relevant in patients with AF. High-risk patients, especially those with chronic diarrhea or disordered defecation, require enhanced monitoring and targeted care, including vigilant tracking of bowel habits and timely medical assessment. These observations generate the hypothesis that promoting regular bowel habits and modulating intestinal motility and microbiota might be relevant to reducing anal crypt infection. This hypothesis warrants prospective evaluation before it can be considered as part of risk management strategies for AF.

This study offers preliminary evidence linking bowel dysfunction to AF risk, providing novel insights for clinical prevention and management strategies. Nevertheless, several limitations warrant consideration. First, the retrospective case–control design identifies associations but does not allow causal inference. Second, this was a single-center, hospital-based study, and controls were drawn from hospital-based inpatients and outpatients rather than the general community population. Thus, Berkson’s bias and limited generalizability cannot be excluded. Third, although data were primarily extracted from electronic medical records, some exposure histories were supplemented by structured telephone interviews when records were incomplete, which may introduce recall bias and exposure misclassification. Fourth, incomplete data on several clinically relevant confounders, including diabetes status, recent antibiotic exposure, prior gastrointestinal infection, and use of stool-regulating medications, precluded full adjustment, leaving the potential for residual confounding. Fifth, the absence of colonoscopic evaluation could have resulted in undetected underlying intestinal disease. Finally, subgroup analyses by fistula level and complexity were exploratory and may have been underpowered to detect modest heterogeneity across subtypes. Future multicenter prospective studies with standardized, objective gastrointestinal assessments and barrier/microbiome evaluations are warranted to validate these findings.

## Conclusions

In this retrospective case–control study, antecedent bowel dysfunction was significantly associated with the occurrence of AF, with chronic diarrhea showing the strongest association, whereas the association with constipation was not statistically significant and warrants confirmation in larger cohorts with refined phenotype definitions. No consistent subtype-specific pattern was observed, although diarrhea exhibited modest heterogeneity across anatomical classifications in exploratory analyses. Once AF is established, fistula complexity may be influenced more by the depth of infectious spread and local anatomic factors than by bowel dysfunction; however, this interpretation remains inferential. In addition, BMI demonstrated a dose–response relationship with AF risk, implicating metabolic and low-grade inflammatory pathways in pathogenesis. These findings may support further evaluation of bowel function as part of future AF risk stratification research. From a clinical standpoint, surgery remains the cornerstone of treatment. Whether proactive “upstream” management of bowel dysfunction might complement surgical care by informing future prevention strategies remains to be determined and requires confirmation in prospective studies.

Future research should prioritize large-scale, multicenter prospective studies that incorporate detailed microbiota profiling and inflammatory biomarker analysis to elucidate the underlying biological mechanisms linking bowel dysfunction to AF. Interventional studies targeting bowel function, dietary patterns, and lifestyle behaviors may further clarify their potential role in AF prevention. Whether incorporating bowel function management into comprehensive strategies could inform future AF prevention is a hypothesis that requires prospective testing; no preventive recommendations can be drawn from this retrospective study.

##  Supplemental Information

10.7717/peerj.21466/supp-1 Supplemental Information 1 Dataset

## Additional Information and Declarations

### Data Availability

The following information was supplied regarding data availability: The data is available in the Supplemental File.

### Clinical Trial Registration

The following information was supplied regarding Clinical Trial registration: no
